# Using Participatory Risk Mapping (PRM) to Identify and Understand People's Perceptions of Crop Loss to Animals in Uganda

**DOI:** 10.1371/journal.pone.0102912

**Published:** 2014-07-30

**Authors:** Amanda D. Webber, Catherine M. Hill

**Affiliations:** 1 Anthropology Centre for Conservation, Environment and Development (ACCEND), Oxford Brookes University, Oxford, United Kingdom; 2 Bristol Zoological Society, Clifton, Bristol, United Kingdom; SUNY College of Environmental Science and Forestry, United States of America

## Abstract

Considering how people perceive risks to their livelihoods from local wildlife is central to (i) understanding the impact of crop damage by animals on local people and (ii) recognising how this influences their interactions with, and attitudes towards, wildlife. Participatory risk mapping (PRM) is a simple, analytical tool that can be used to identify and classify risk within communities. Here we use it to explore local people's perceptions of crop damage by wildlife and the animal species involved. Interviews (n = 93, n = 76) and seven focus groups were conducted in four villages around Budongo Forest Reserve, Uganda during 2004 and 2005. Farms (N = 129) were simultaneously monitored for crop loss. Farmers identified damage by wildlife as the most significant risk to their crops; risk maps highlighted its anomalous status compared to other anticipated challenges to agricultural production. PRM was further used to explore farmers' perceptions of animal species causing crop damage and the results of this analysis compared with measured crop losses. Baboons (*Papio anubis*) were considered the most problematic species locally but measurements of loss indicate this perceived severity was disproportionately high. In contrast goats (*Capra hircus*) were considered only a moderate risk, yet risk of damage by this species was significant. Surprisingly, for wild pigs (*Potamochoerus* sp), perceptions of severity were not as high as damage incurred might have predicted, although perceived incidence was greater than recorded frequency of damage events. PRM can assist researchers and practitioners to identify and explore perceptions of the risk of crop damage by wildlife. As this study highlights, simply quantifying crop loss does not determine issues that are important to local people nor the complex relationships between perceived risk factors. Furthermore, as PRM is easily transferable it may contribute to the identification and development of standardised approaches of mitigation across sites of negative human-wildlife interaction.

## Introduction

Quantifying crop damage has a fundamental role in determining loss yet does not provide a complete or accurate representation of the impact of crop damage by animals on affected communities. For example, the economic loss sustained by wildlife can also result in substantial social costs, including reduced food security, health care, education, labour, land tenure, access to resources and psycho-social well-being [1], [2], [3], [4], [5]. These costs contribute to negative attitudes towards animals and an increased perception of risk, reducing tolerance for wildlife and impeding the success of conservation initiatives [6], [7], [8], [9]. Conversely, the study of attitudes reveals that people do not always perceive a problem with wildlife that forage on their crops and may be willing to tolerate such losses when they occur [10],[11]. Consequently, crop loss mitigation is not always appropriate, may focus upon a ‘conflict’ that does not exist [12], [13], or even perhaps precipitate a conflict situation where previously there was none. Therefore, understanding people's perceptions of the risk of crop damage by wildlife and what influences those perceptions is central to understanding the impact of crop damage by animals on local people.

Risk can be defined as “exposure to potentially unfavourable circumstances” ([14], p. 1946) and, in the context of this paper, a negative impact upon crop yield. “Intuitive risk judgements” ([15], p. 280) or perceptions of risk, are complex and variable; they can differ within communities and conflict scenarios [16] and may not reflect assessed risks but be a “surrogate for other social or ideological concerns” ([15] p. 285). For example, attitudes toward animals and their utilization of human foods may not reflect measured crop loss. Wild species are often reviled for causing damage even though domestic animals are responsible for a high proportion of the loss [17], [18]. Additionally, specific species may attract attention due to their large body size, gregarious nature or potentially dangerous behaviour; elephants, primates and carnivores often attract a disproportionate level of blame [2], [19], [20]. This can result in the persecution of wildlife [12] and even erroneous crop protection that can increase the density of certain agricultural ‘pests’ [21].

The reasons for the disparity between assessed and perceived loss are not always clear. Farmers may simply misidentify the damaging species or misunderstand its relationship with domesticated crops [22], [23]. Furthermore, perceptions toward animals may not be a reflection of damage but rather a social tension or a symbolic threat [24], [25], [26]. Complaints regarding wildlife adjacent to protected areas can reveal discontent with conservation legislation and subsequent limitations in access to resources [25], [26]. To manage or mitigate conflicts around wildlife it is important to understand not only whether local people perceive a problem, and if so what it is, but also to explore factors that may influence their views [27], [28]. This is vital as risk perceptions are persistent (even in the face of contradictory empirical evidence) and they can impact upon the acceptance of future information [15], [29]. It is also not appropriate to rely on measures of assessed risk to mitigate conflict as they may not address the true reasons for people's behaviour [16]. Indeed, research shows that it is social and psychological factors that drive retaliatory killing of ‘conflict’ animals and not levels of damage [26].

Here we adopt a technique called participatory risk mapping (PRM), first developed by Smith et al. [14] to order and classify sources of risk faced by livestock producers in East Africa. It was further tested by Quinn et al. [30] who examined perceptions of risks to livelihood more widely to include pastoral and agricultural communities in Tanzania. More recently, PRM has been used by a small number of researchers interested in human-wildlife interactions. For example, Baird et al. [16] explored the impact of a National Park on risk perceptions in Tanzania and Inskip et al. [28] examined perceptions of the risk of a single wild animal species (tigers) compared with other livelihood risks in Bangladesh. Kahler et al. [31] also used PRM to create spatial maps of the perceived risk of poaching in Namibia (these were compared with poaching events using Geographic Information Systems, GIS). Using data from interviews and focus groups, we use PRM to identify and rank risk factors that farmers consider detrimental to their agricultural productivity, including wild animals. We then extend the method to compare perceived risk of crop loss to particular animal species against measured losses using the same approach. We demonstrate how PRM can contribute to our understanding of human-wildlife interactions at the local level; perceived and assessed risk are not analogous at this site and this simple tool highlights areas of potential conflict and mitigation. This extension to current PRM methods could have significance for those who require a holistic understanding in order to develop effective mitigation, targeting farmer priorities, particularly where time and funding resources are limited. To our knowledge, this is the first study to use this effective and easy to use tool to examine the relationship between measured and perceived risk in a human-wildlife interaction scenario.

## Methods

### Ethics statement

Ethics clearance was granted by Oxford Brookes University Research Ethics Committee prior to beginning data collection (including the specific methods adopted for obtaining and recording informed consent). Only those people who gave verbal consent were included in the study and their personal information was recorded separately to maintain anonymity during analysis and reporting. The procedure adopted for ensuring informed consent in a predominantly illiterate population, where the culture is based primarily on an oral tradition, entailed explaining the purpose and scope of the research verbally to each farmer, outlining what participation would entail, and giving them time to reflect on this information and ask questions of the research team, prior to giving their consent to participate. It was also stressed to potential participants that participation was voluntary and they could withdraw at any time. Individuals' consent to participate was recorded in writing by AW because the use of a digital voice recorder was considered overly intrusive. Consent was renegotiated with each participant at every stage of the project as recommended by the Association of Social Anthropologists [32], and a written record of their willingness to participate kept by AW.

### Study site

**Figure 1:**
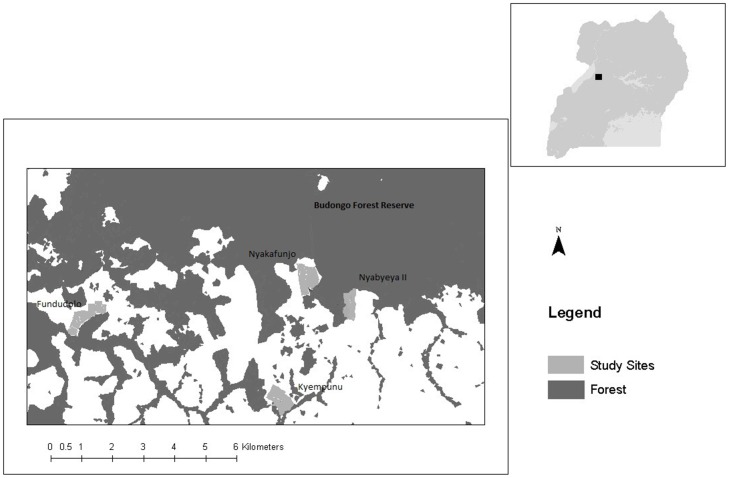
Map showing the location of the four study areas in relation to Budongo Forest Reserve (inset Uganda country map). Adapted from Webber et al. 2007 [63].

The study was carried out in 4 villages (Kyempunu, Nyabyeya II, Fundudolo, Nyakafunjo) at the southern edge of the Budongo Forest Reserve (BFR), Uganda (Figure 1). These villages, and associated agricultural areas, were selected because of their proximity to animal habitat; all bordered a forest fragment or BFR so were likely to experience wildlife incursions. A strip of land (1 km×0.5 km) next to the forest edge was identified in each village and all farmers within these areas were invited to participate in the study, after Naughton-Treves [17]. Only one farmer in 2005 indicated they wished to withdraw from the research project; all data relating to them were excluded from the analysis. The final sample comprised 129 farms (total of 169 fields).

This was an economically poor and vulnerable population [1], [33]. Approximately one third of the study sample was over 46 years old and 26% had no formal education. Almost 70% were dependent on farming as their main source of income; despite this mean farm size was particularly small at 0.65 ha [34]. Twenty different ethnic groups were represented within the sample although only 4% were from the native Banyoro [34]. Over half of the participants (53%) were from the West Nile/Northern Uganda region and this reflects migration due to employment opportunities and civil unrest in these areas [1]. It also suggests that migrants were disproportionally represented on the forest edge as seen at other sites [35].

Data were collected by AW and three Field Assistants (FAs) for 13 months between February 2004 and November 2005 to coincide with growing seasons. The FAs (2 male, 1 female) were chosen to reflect a range of ethnic backgrounds, age and gender to enable the study to work as efficiently in the local community as possible. The Lead Researcher (AW) is female, ensuring a gender balanced research group. All three FAs were recommended to AW and then employed for a trial period to assess their suitability for the role. They all had farming experience and lived locally, although not in the 4 study villages. This ensured they were familiar with the area but potential conflicts of interest were reduced. The three FAs were, between them, fluent in all the main languages spoken locally including KiSwahili, Runyoro, Lugbara, Alur and Okebu. This was essential as it soon became apparent that the KiSwahili learnt by AW in the UK was very different from the local version in use at the study site. To integrate into the community, the research team travelled by foot or bicycle (the most common modes of transport locally), ate in local markets and spent time in farms participating in agricultural tasks (e.g. hoeing and weeding). Inevitably, there were potential sources of bias associated with the presence of an international researcher and the use of local FAs. However, extended interaction with the local community during 13 months of fieldwork, especially time spent in the villages outside of systematic data collection activities, combined with AW adopting a reflexive approach, helped minimise potential sources of bias within the data sets collected.

### Monitoring crop losses

Study farms were surveyed at weekly intervals during the periods March to end of July 2004 (study season 1), March to end of July 2005 (study season 2) and August to November 2005 (study season 3) to monitor crop damage by wild and domestic animals of 2 kg or above (large vertebrates). AW and/or the three FAs systematically walked around and through each crop within each field taking care to scan and record crop damage events but not to damage standing crops.

The total number and type of crops grown were assessed for each field (N = 169) and recorded during each study/growing season. Damage was measured directly in m^2^ for crops with dense groundcover i.e., beans and groundnuts. Average planting density was calculated for all other crops [35] by selecting 4 random samples of each crop and obtaining the mean number of stems grown (or heaps for sweet potato) in a 100 m^2^ area. Damage for these crops (e.g., maize, cassava, sweet potato) was measured by counting damaged stems or heaps and converting to m^2^ using average planting densities. Externally visible plant parts/planting regimes were counted (i.e., stems and heaps rather than tubers) for greater accuracy and ease of recording. Spoor, dental impressions, scat and damage patterns were used by the research team to identify species causing crop damage. The Field Assistants were trained by AW but had each been farming for a long time and were experienced at identifying crop damage and the animal responsible. To ensure inter observer reliability, the research team worked in pairs to record crop damage. Both individuals had to agree on the animal causing damage and AW rotated around each pair. At least 2 pieces of secondary evidence were required to confirm identification of animal species; where this was not available, or crop damage was more than 1 week old, that damage event was not assigned to a particular animal species. The presence of damage by other factors (weather, insects, termites and people) was also recorded.

### Identifying Perceived Risks: Semi-Structured Interviews

Semi-structured interviews (SSIs) with farming households included in the crop damage survey were initially conducted during April through to June 2004. One adult representative from each household/farm (the individual most frequently encountered in the field) was invited to participate (n = 93 as some farms were not regularly attended by farmers). Follow up interviews were completed during August through to November 2005 (n = 76) to further examine perceptions. Every effort was made to use the same individual for both interviews but where this was not possible (11), another household member was approached. Demographic information was recorded for all 104 interviewees (Male = 62, Female = 42).

Interviews were conducted in farmers' fields or homes with the help of a FA as translator. Responses were noted by the research team as voice recordings were considered too invasive for this research. Much has been written on the limitations of using translators within social research [36]. However, with multiple languages in the study sample, translators were essential to the success of this project. Pilot interviews and weekly meetings of the research team were conducted to ensure that research concepts and relevant cultural knowledge were explored between team members (e.g., myths and the utilization of trees/animal species by different ethnic groups). These elements were also discussed with other bilingual local people to confirm the validity of the data collected.

During SSIs, farmers were asked to list all the problems they could or might experience with their crops. Responses were translated into English and organised into categories retaining distinctions made by the participants [14]. As Smith et al. [14] and Inskip et al. [28] highlight, it is important to choose local words that accurately reflect the concept of subjective risk. Therefore, language was discussed in depth among the research team in advance of the interviews to ensure that all translation expressed the concepts of ‘anticipated problem’, ‘concern’ and ‘worry’. For clarity we will use the term ‘risk’ throughout this paper. Where farmers identified crop damage by animals as a ‘risk’ they might experience, they were then asked to list the various species involved. This was asked before any specific questions examining crop loss to avoid leading the participant [28]. There was no limit to the number of responses people could give, therefore the number of ‘risks’ varied for each interviewee.

To understand the attitudes of local people towards specific animals more fully, interviewees were also asked to assign one word to describe different animals (baboons, wild pigs, monkeys, bush duikers, goats and chimpanzees). The species were selected by the researcher as they either cause the most damage in fields in this area or they have high conservation significance i.e., chimpanzees (*Pan troglodytes schweinfurthii*) [34], [37], [38]. The selected words were translated and categorised by the research team. Due to the inherent difficulty in translating these concepts across multiple local languages, this was discussed at length. Having FAs from a range of different ethnic backgrounds with knowledge of a multitude of different languages and the appropriate cultural context was invaluable at this stage.

It should be noted that several species of primate (blue monkey - *Cercopithecus mitis stuhlmanni*, red-tailed monkey - *Cercopithecus ascanius schmidti* and black and white colobus - *Colobus guereza occidentalis*) were combined into the category of ‘Monkey’ for data analysis. Picture cards were used in the early stages of this research project and this helped to ascertain how these animals were considered and distinguished linguistically by local people; while the majority could distinguish between monkey species this was not usually done unless they were asked specifically to identify them individually. Additionally, it was very difficult to differentiate between secondary damage evidence for monkey species. Therefore, lumping these three species was appropriate for this study. Clearly baboons (*Papio anubis*) are also monkeys from a taxonomic perspective but their terrestrial behaviour meant that local people responded very differently to them [18]. It was therefore appropriate to consider baboons in a category of their own.

### Focus Groups

Additional information was collected in 7 focus groups; participants were randomly sampled from within each study area (n = 31). As Bloor et al [39] note, recruiting small numbers of people to groups does increase the risk of poor attendance and limited interaction. However, using a small number of participants can be more useful as a natural setting for discussion. Focus groups were used to contextualise information and explore issues raised in the SSIs in more detail to gain a better understanding of the perceived risk of crop damage by animals when compared with other threats to crop yield. Focus groups also provided a useful opportunity to explore attitudes towards different animal species. A structured topic guide was used in this study to allow for a consistent approach in both delivery and the final analysis [40], [41]. Men and women worked in separate groups to encourage open and honest discussion; men are generally the dominant gender in rural Uganda and it was important to ensure that women could express their views without fear of intimidation or retribution [42], [43]. Focus group data were pooled as this paper is concerned with providing general perceptions and group norms to support the SSI results as opposed to a detailed examination of gender differences. FAs assumed the role of Facilitators and the sessions were translated immediately and points noted by AW and/or another FA. Using ‘indigenous researchers’ as facilitators had the advantage of (i) relaxing the participants, (ii) potentially encouraging more honest responses, and (iii) enabling the FAs to gain valuable research skills [39]. There are inevitably some limitations with FAs assuming this role, e.g. over familiarity with participants and cultural hierarchies between individuals, but the team frequently discussed this issue in order to try and minimise its impact.

### Risk Maps and Indices

To analyse the ‘risks’ that local people identify as threatening crop productivity, risk maps were produced as per Smith et al. [14] and Quinn et al. [30]. A severity index was calculated for each risk as follows, *Sj* = 1+(r−1)/(n−1), where r is the rank based on the order of response by the interviewee and n the total number of risks listed by that respondent [30]. The mean distribution was calculated for all respondents who highlighted the risk and this created a score from 1 (most severe) to 2 (least severe). An incidence index (*Ij*) was created to measure the proportion of respondents stating a particular risk; this score ranges from 0 (not mentioned) to 1 (mentioned by all). By dividing incidence by severity a risk index was created (*Rj*); the higher this figure, the larger the perceived risk of the problem.

Additionally, risk maps were produced to compare farmers' perceived risk of crop damage by specific wildlife species with measured crop damage caused by those same species. To do this a severity index was developed for each farm that experienced crop damage; r was based on the rank of area damaged in each farm by a particular species (within each farm species were ranked by the area they damaged) and n was the total number of animal species recorded damaging crops in that farm. The incidence index reflects the proportion of farms experiencing crop damage by each species. The perceived risk of specific animal species was calculated from interview data using the method outlined above. A severity index, *Sj* = 1+(r−1)/(n−1) was created where r was the rank based on the order (and thus magnitude of risk) of each species by the interviewee and n the total number of species listed by that respondent [30]. The mean distribution was then calculated for all respondents who highlighted the risk and this created a severity score. An incidence index (*Ij*) was created to measure the proportion of respondents stating that a particular species damaged their crops. As above, the risk index was created by dividing incidence by severity. Perceived and assessed risk indexes were then plotted on the same graph to give a visual representation of any similarities or differences and the direction of variance.

## Results

### Measured Crop Damage

A number of different sources of crop damage were recorded in farms around BFR; large vertebrates (e.g., primates and ungulates), insects, termites/stemborers, birds, disease, weather, people and small vertebrates. Eighty percent (n = 104) of farms experienced at least one damage event by large vertebrates but this was not the most frequently recorded cause of crop damage. Evidence for insect damage (including crickets, grasshoppers and caterpillars) was found in 82% of farms studied (n = 106). As farmers categorised insects by the type of damage they caused, termites and other stem borers were considered separately; their stem damage was seen in 74% of farms (n = 96). Damage patterns by birds meant farmers also considered them differently to other wildlife, consequently we categorised them separately; more than a third of farms had evidence of bird damage to crops. In addition, crop disease (including cassava mosaic, maize streak and banana wilt) was observed in 42% of farms (n = 54).

Evidence of crop damage caused by extreme weather was a feature of 39% of farms (n = 50) and included hailstones, strong winds, flooding and drought. Perhaps the most surprising damage was due to people; 28% of farms (n = 36) experienced crop loss as a result of careless weeding, out of control fires, children playing and flattening by vehicles.

Crop damage by large vertebrates was a common problem in this area; 689 damage events were recorded and a total of 6093.7 m^2^ of agricultural crops damaged over the three field seasons. Eleven large vertebrate species damaged cultivated crops at the study site (Table 1). Wild animals were responsible for more than half of all recorded damage events (n = 394, 57%) and over 62% of area lost (3791.36 m^2^). Baboons were responsible for 33% of the area damaged at this site (2059.5 m^2^). Monkeys also foraged in crops (13%) but only 212.1 m^2^ of damage was due to these animals. Twelve percent of all crop damage events and 16% of the total area lost was attributed to wild pigs (*Potamochoerus sp*.). Damage by chimpanzees was rarely recorded. Domestic species were responsible for 41% of all damage events (n = 282), with goats (*Capra hircus*) causing the largest proportion of this loss (36% of crop raids and 1664.6 m^2^ of damage).

**Table 1:** Frequency of crop damage events and area damaged (m^2^) by animal species (bold figures indicate the highest rank for that category).

*Crop Foraging Species*	*Latin Name*	*Damage Events (Freq)*	Area Damaged (m^2^)
*Wild*		*394*	*3791.36*
Bush Duiker	*Sylvicapra grimmia*	35	353.5
Monkey	*Cercopithecus & Colobus sp.*	92	212.1
Baboon	*Papio anubis*	164	**2059.5**
Chimpanzee	*Pan troglodytes schweinfurthii*	13	8.9
Wild Pig	*Potamochoerus sp.* 1	83	1006.8
Buffalo	*Syncerus caffer*	4	137.36
Porcupine	*Hystrix cristata*	3	13.2
*Domestic*		*282*	*2033.6*
Goat	*Capra hircus*	**250**	1664.6
Domestic Pig	*Sus scrofa*	19	123.8
Cow	*Bos taurus*	12	244.5
Sheep	*Ovis aries*	1	0.7
Pig^2^		13	269
**Total**		689	6093.7

1 Previous studies refer to the presence of *P. porcus* in this area [1], [43] although it has been suggested that it should be *P. larvatus* at the edge of its range [62]. It is not possible to verify the taxonomy of this species as no wild pigs were observed during the study.

2 Secondary evidence at the crop damage site confirmed the animal responsible to be pig; however, in these cases it was not possible to determine whether it was a wild or domestic species.

### Perceptions of Crop Damage Proportional to Other Risks

Interviewees (n = 93) listed a series of different risks to their crops that we later grouped into 11 categories to create a risk index (Table 2). These included crop raiding wildlife, insects, poor soil and lack of land. (We use the term ‘crop raiding’ here only because this is how these activities are referred to locally and more widely in Uganda). Crop damage by wildlife had a much higher risk index than other risks in all villages. Termites and poor weather conditions were also considered a risk around BFR.

**Table 2:** Risk index of perceived problems experienced with crops; bold figures denote the highest ranking issue; X indicates no response (159 responses); high index values indicate risks perceived as most significant.

	*Total Risk Index*	*Kyempunu Risk Index*	*Nyabyeya II Risk Index*	*Fundudolo Risk Index*	*Nyakafunjo Risk Index*
Crop Raiding Animals	**0.702**	**0.693**	**0.732**	**0.743**	**0.630**
Termites	0.125	0.250	0.129	0.070	0.123
Insects	0.072	0.112	0.112	0.055	0.058
Weather	0.120	0.113	0.213	0.055	0.116
Poor Soil^1^	0.062	0.083	0.112	0.035	0.023
Weeds	0.015	X	X	0.015	X
Birds	0.007	0.041	X	X	X
Land Ownership^2^	0.006	X	X	X	0.015
Lack of Land	0.013	X	X	0.015	0.020
Planting Strategies	0.006	X	X	X	0.035
Thieves^3^	0.022	X	X	0.03	0.03

1 Too many stones, poor quality, marrum (laterite) soil, over use.

2 Not experienced by crops per se but farmers referred to this to describe concern with unofficial, indistinct and, in some cases, temporary land use agreements.

3 Included in this category are people who take food crops or sugar cane from farms without the owner's permission.

**Figure 2:**
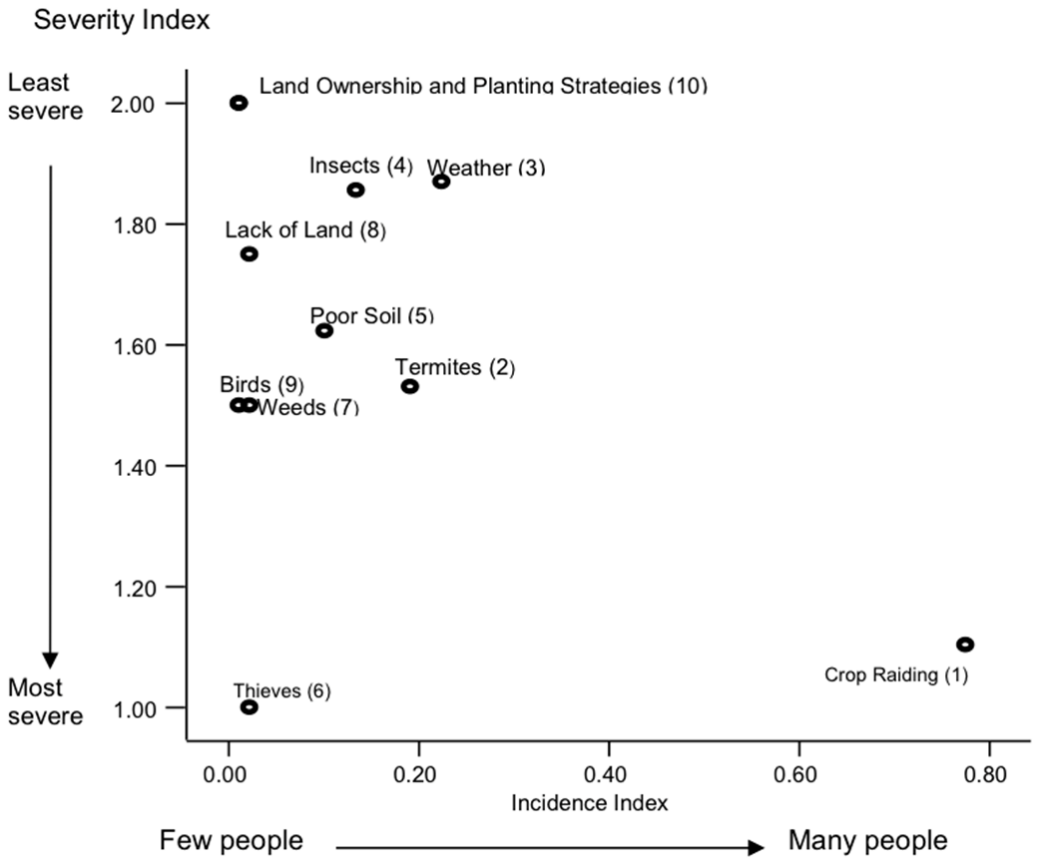
Risk map overview depicting farmers' perceptions of crop damage by animals proportional to other risks to agricultural production (159 responses). Severity is measured from 1 (most severe) to 2 (least severe). Risk index rank is in parenthesis.

A risk map overview was created (Figure 2) to demonstrate graphically the relationship between frequency and severity of response. Crop damage by wildlife stands apart from other perceived risks because it has a high severity index (*S* = 1.103) and the highest incidence index of all issues (*I* = 0.775). By contrast, thieves score highly on the severity index (*S* = 1) but only a few farmers perceive them to be a problem (*I* = 0.022). It is important to note here that during discussions of general risks experienced by farmers, domestic animals, and specifically goats, were not identified as a significant cause of crop loss.

### Perceptions of Specific Animal Species

**Figure 3:**
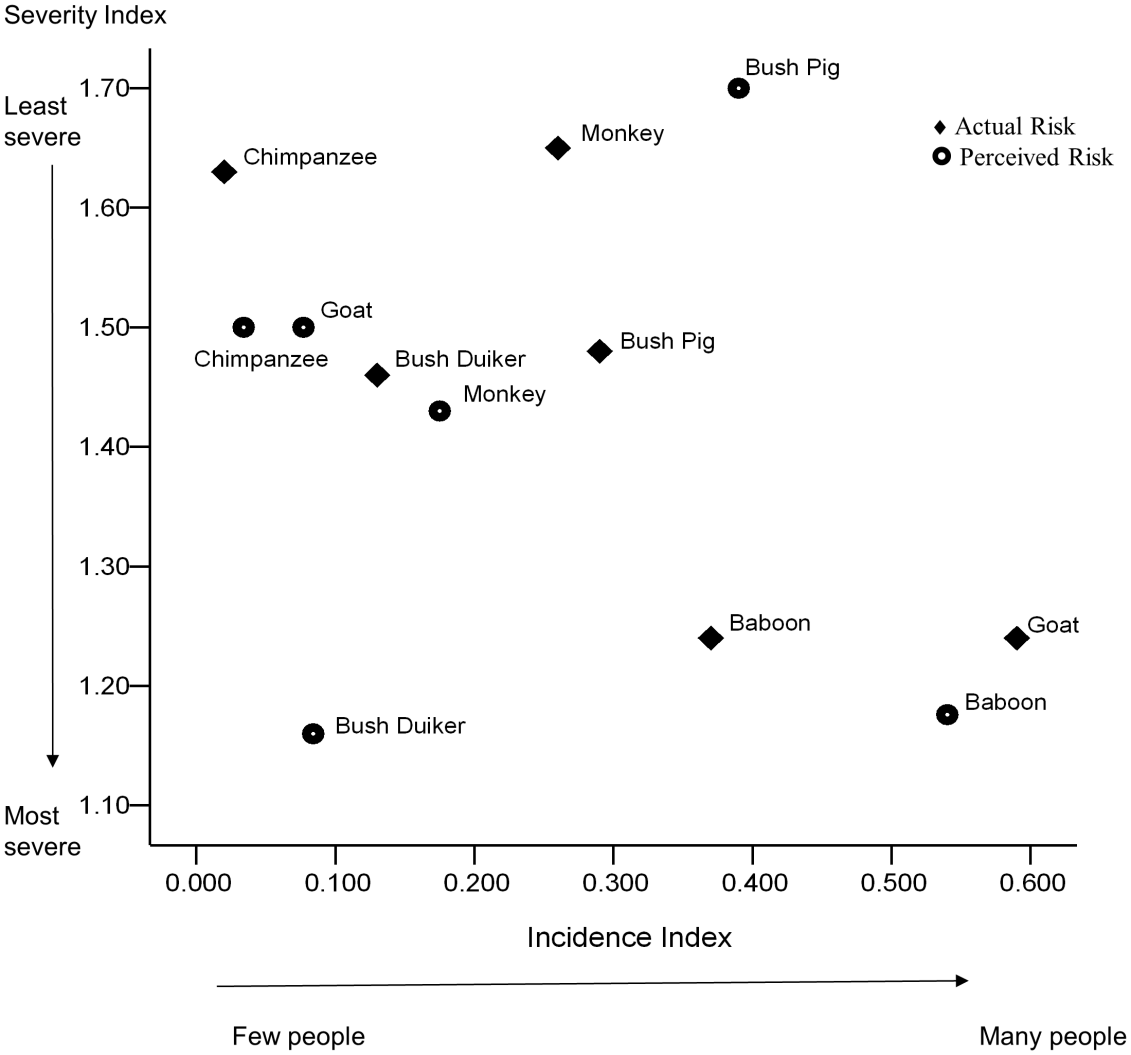
Risk map overview depicting farmers' perceptions of problem animals (132 responses) and assessed damage by those species (98 responses). Severity is measured from 1 (most severe) to 2 (least severe).

Baboons were considered the most problematic species within the study (Table 3). Many people described them as the ‘enemy’ and they were perceived to be highly destructive with negative character traits; ‘greedy’, and ‘thief’. Farmers discussed how ‘baboons will destroy even what they do not eat’ and that they ‘come just to spoil not to eat’. Perceived severity and incidence indexes were higher than assessed risk, suggesting local people believe baboons are a greater risk than analysis of damage (proportional to other species causing crop damage) demonstrates. Wild pigs were also mentioned by many interviewees but were regarded as less problematic than baboons (Table 3/Figure 3). During SSIs they were described as the ‘enemy’ but to a lesser extent; most words used (‘destroyer’, ‘grader’, ‘sweeper’ and ‘tractor’) refer to the manner in which wild pigs damaged farms. Surprisingly, perceptions of severity were not as high as recorded damage, although perceived incidence was greater than frequency of damage events recorded (Table 3).

**Table 3:** Assessed and perceived risk index for key animal species.

*Species*	*Perceived Severity Index*	*Assessed Severity Index*	*Variance*	*Perceived Incidence Index*	*Assessed Incidence Index*	*Variance*
Baboon (*Papio anubis*)	1.17	1.24	+P	0.54	0.37	+P
Wild Pig (*Potamochoerus sp.*)	1.70	1.48	+A	0.39	0.29	+P
Monkey (*Cercopithecus & Colobus spp.*)	1.43	1.65^1^	+P	0.17	0.26^1^	+A
Bush Duiker (*Sylvicapra grimmia*)	1.16	1.46	+P	0.08	0.13	+A
Goat (*Capra hircus*)	1.50	1.24	+A	0.07	0.59	+A
Chimpanzee (*Pan troglodytes schweinfurthii*)	1.50	1.63^1^	+P	0.03	0.02^1^	+P

+ indicates the highest factor (i.e., A = assessed or P = perceived risk). Severity index measured from 1 (most severe) to 2 (least severe) and incidence from 0 to 1 (most reported).

1 Assessed severity and incidence indexes are conservative for chimpanzee and monkey due to fruit and sugar cane not being included in estimates of damage area.

Monkeys were described as ‘thieves’ because they were observed ‘peeping’ and ‘hiding’ in vegetation, particularly near farm boundaries. Words used during SSIs to describe monkeys were positive considering many farmers were referring to crop damage experiences; monkeys were described as ‘clever’ and ‘faithful’. However some people referred to monkeys as ‘destroyer’ and ‘bad character’ due to their destruction of food crops and a few respondents perceived monkeys to be a moderate risk to agricultural productivity (Table 3/Figure 3). PRM highlighted that bush duikers were considered to be a severe risk by a few people. Words chosen to describe bush duikers (*Sylvicapra grimmia*) ranged from ‘good character’ and ‘clever’ to ‘enemy’. They were also regarded as intelligent because they are ‘fast’, ‘difficult to trap’ and ‘selective’ in the crops they damage. Monkeys and bush duikers also had a higher rate of assessed rather than perceived incidence but for severity the results were reversed; perceptions of loss were greater than observed crop damage, proportional to other species.

Chimpanzees were considered to be a moderate risk by several farmers but perceptions of this species were predominately positive (Table 3/Figure 3). Participants in SSIs described them as ‘human’, ‘humble’ and ‘respectful’, considered to ‘walk gently’ and be ‘disciplined’ in their crop damage, only removing as many food items as they need. However, some farmers did fear chimpanzees, describing them as ‘friendly but dangerous’. Interestingly, the only time chimpanzees were considered ‘destroyers’ was if the interviewee was discussing sugar cane. If measured loss to chimpanzees was indeed under-represented in this study, assessed and perceived risk appears very similar for this species (see Table 3).

Goats were considered a moderate risk by interviewees. They were perceived in a very different way to wild species; damage by this ‘little destroyer’ as they were referred to during SSIs, was considered the result of negligence on the part of the owner (‘home property’ and the ‘farmer’s responsibility’). Goats had the most significant variation between assessed and perceived risk; area damaged and the number of farms experiencing damage to this species were proportionally much higher than local people stated (Figure 3/Table 3).

## Discussion

### Crop Damage as an External Issue

Crop damage by wildlife was one of many risks people reportedly experience with their crops. Insects, poor weather conditions and lack of land were also believed to impact negatively on rural livelihoods. Farm monitoring confirmed crop damage by wildlife was a significant problem in the study farms; however it was not the most prevalent issue and insect damage was recorded more regularly. Despite this, farmers considered crop damage by wildlife to be a more significant risk than other factors. This is consistent with other studies [1], [23], [44], [45] and suggests that farmers are less tolerant towards crop damage by wildlife. Understanding this disparity is vital for those engaged in conflict mitigation.

Like damage from termites, birds, thieves, and weather, crop damage by large vertebrates can be defined as an ‘external’ agricultural issue, and an outside influence that physically enters and manipulates the agricultural domain. In contrast, soil fertility, planting strategies and farming methods are ‘internal’ problems that originate and proliferate from within the farm boundary. In this study, external agricultural issues were perceived as more severe than those internal to the farm, as has also been reported in other studies around BFR. For example, Tweheyo et al. [33] found that poor sowing and fire (usually started by the farmer from within the field) were considered to be less problematic than wildlife, drought and insects.

One of the most pervasive determinants of a high perception of risk, as outlined in Fitchen et al. is if the threat is believed to be external to the community [46]. This has also been found at other sites of conservation conflict; for example, jaguars were perceived to be a problem in Brazil where farmers believed, incorrectly, they were being introduced to the forest [47]. This heightened level of perceived risk regarding damage by wildlife may be because external agricultural problems are more difficult for the farmer to manage than internal issues. For example, nocturnal animals are often tolerated less than diurnal species because farmers cannot adequately protect against depredations [7], [17]. Furthermore, despite significant damage, goats in this study were only considered a moderate risk. This could be due to the animal's economic and cultural importance or because farmers perceive themselves better able to manage damage through tethering the animals away from crops or through institutional methods of compensation [17], [25], [48]. This contrasts with damage from wildlife which is not compensated and is influenced primarily by proximity to a protected area. Frustration at this additional, unfair, burden [2], and a perceived inability to manage crop damage, may raise risk perceptions and reduce tolerance for wildlife at this site.

### Crop Damage as an Associated Issue

Alternatively, or additionally, crop damage by wildlife may be perceived as a particularly severe risk around BFR because of its association with other issues that can limit agricultural yield, in addition to social and economic development, as found by Inskip et al. [28]. For example, loss of food can cause famine and lack of income resulting in poor health, fewer educational opportunities and a delay in community level development.

Focus group discussions also indicated that many risks were intrinsically linked with one another. For example, lack of land was given as a reason for planting crops next to the forest edge. Land shortages force local people to adopt high risk planting strategies especially if other areas of the farm have become exhausted from intensive agricultural use; soil fertility was perceived to be better at the forest margin. Farmers were aware of the increased risk of crop damage by wildlife through this practice although, surprisingly, many considered it prudent to plant maize in these areas. They stated that as a seasonal crop, maize was only vulnerable for short periods of the year (8–12 weeks) and guarding intensity could be increased accordingly. However, if cassava were grown at the forest edge rather than closer to the house it would require guarding for 18 months or more, which was beyond the capabilities of the average household. It highlights the importance of understanding if and how risks are linked together as opposed to considering them as isolated entities [14], [16], [28].

### Perceptions of Specific Animal Species

Chimpanzees were the only species in this study to have comparable assessed and perceived risk results and be considered in a predominately positive manner. However, some respondents were afraid of chimpanzees and were reluctant to adopt crop protection strategies due to their possible aggressive behaviour, as has been seen elsewhere [49]. This is a potential concern for conservation locally because people are likely to become intolerant of a situation they cannot control. It is particularly relevant for those growing sugar cane as the potential for greater economic loss appears to have created negative attitudes toward this endangered primate [50], [51].

Perceptions of other crop foraging species were more mixed. For example, views of baboons and wild pig were very negative amongst this sample living and/or working on the forest edge, as noted around Kibale National Park, Uganda [5]. These farmers were generally more vulnerable from incursions by wild species [37]. However, as mentioned previously, goats were not perceived to be a problem and yet many farms experienced damage from these animals [52]; indeed the biggest variation between assessed and perceived loss occurred for this species. This could be due to comparatively inconspicuous damage patterns (goats eat predominately leaves) and their importance economically and culturally [52]. It could also reflect the fact that local people perceive goats to be the ‘farmer's responsibility’ therefore easier to control than wild species. Value judgments appear to be influenced by a complex combination of variables.

Body size is important when examining perceptions of species causing crop damage [2]. Large animals tend to be blamed disproportionately for crop damage [7], [53], [54], perhaps because they are visually intimidating, and can cause considerable amounts of loss [55]. Large animals are also an obvious and detectable risk unlike ‘invisible’ threats to human health, for example, water contaminants [46]. However, body size does not adequately explain the differential perceptions of crop foraging species at this site. For example, chimpanzees are the largest primate in this study and yet were perceived more positively than baboons. It is possible this view was due to knowledge of their protected status. However, the language used to describe this species suggests it is more to do with an anthropomorphised understanding of their feeding behaviour [18].

Chimpanzees foraging on food crops were described as behaving in a considerate manner, only taking what they needed. Bush duikers were also considered ‘respectful’ and ‘disciplined’ in the way they utilized human foods. Both species were believed to control the amount and part of crops taken, only removing fruits or leaves as required. This was in sharp contrast to the negative perceptions of wilfully ‘greedy’ baboons and ‘destructive’ wild pigs It is interesting that farmers underestimate the severity of wild pig damage proportional to other species that caused damage. However, we suggest this is more likely indicative of an extreme response to crop damage by other animals e.g., baboons, rather than a misunderstanding of wild pig damage.

Crop foraging behaviour of monkeys (blue monkey, red-tailed monkey and black and white colobus) also appeared to be more acceptable to local people. The majority of farmers perceived them to be ‘thieves’, a term used in other situations where monkeys and people are in close proximity [44], [56], yet many described them as ‘clever’, damaging crops individually and making quick, opportunistic forays into agricultural areas. This perceived selectivity was tolerated unlike the highly visible damage by baboons and wild pigs. Additionally, as pith eaters, baboons chew the immature stems of crops e.g., maize [37], [48]. By utilizing plant parts that are unsuitable for human consumption yet integral to plant survival to maturity, they are believed to be ‘wasteful’, destroying ‘even what they do not eat’. Similar findings have been reported for other sites and species [24], [35], [57], [58] reflecting ways in which social rules and human values are imposed upon wild animals [18], [59].

### PRM

This study highlights how PRM can help researchers to identify and explore perceptions of risk at sites with potentially negative human-wildlife interactions. The four villages sustained crop damage from a variety of different factors, however quantification of loss alone would be overly simplistic and could have led to ineffective and inappropriate mitigation strategies. For example, while goats are clearly a major problem in this area, focussing on loss to domestic species without understanding perceptions could heighten tension and be interpreted by local people as a diversionary tactic by wildlife authorities [52]. Extending Smith et al.'s method [14] to compare assessed and perceived risks proportionally to one another alongside other qualitative methods allows for a more holistic view of human-wildlife interactions and their perceived relationship with other factors [14], [28]. For example, PRM results from this study identified baboons as a priority concern for local farmers, informing the development of crop protection tools designed specifically to reduce foraging opportunities for baboons on farms [60]. However, as this study has highlighted, wildlife managers and researchers may also need to address associated issues of land availability and ownership [26], [28] alongside livestock husbandry strategies. This will require long term intervention and collaboration across a number of disciplines and agencies.

## Conclusions

PRM provides a simple, but effective tool to explore how people perceive and prioritise the threat of crop damage by wildlife. It is inexpensive, requires little technical knowledge, and can be used across a range of sites and with different literacy levels [28]. Additionally, the results are in a format that are easy to communicate to stakeholders [14]. This paper demonstrates how this very easy to use and cost effective method can help to identify and explore the complexity of the relationship between measured and perceived crop damage. Previous studies that use PRM to examine human-wildlife interactions have focused on exploring perceptions without considering assessed risk [16], [28]. While these studies have value, both elements are required in order to fully understand and potentially mitigate a human-wildlife conflict scenario. Kahler et al. [31] used GIS to examine and compare perceived and documented risk of poaching in Namibia. While using PRM to understand the spatial relationships of risk is very important [14], analysis using GIS frequently requires expensive software and specific analytical skills [61]. We believe the value of PRM lies in its accessibility and we understand that this study is the first time that PRM has been used to compare assessed and perceived risk using the same method.

It is very unlikely that simply reducing crop damage without understanding people's perceptions will lead to conflict resolution [26]. However, when used in conjunction with monitoring of crop damage incurred, this method can help researchers and practitioners understand how local people perceive risks to their agricultural productivity and thus assist in designing effective, targeted conflict mitigation where required [14], [28], [29]. It can also assist in the identification of key stakeholders; seldom can all the affected community be involved in mitigation interventions and practitioners need to ensure that they are engaging the ‘right’ people and not just displacing the problem [26].

PRM also provides an opportunity to make standardised comparisons across sites, to help identify commonalities and differences, as a first step to examining the degree to which different conflict mitigation techniques might be transferable between different sites or conflict scenarios. It is undoubtedly a simple method but one that has potential to contribute significantly to our understanding of human-wildlife relationships and people's perceptions of risks associated with those interactions. Consequently PRM has the potential to be a useful analytical tool for wildlife management and conservation science.
